# Ancient Schwannoma of the Thigh: Metaplastic Ossification, Cartilage Formation, and Extensive Calcification

**DOI:** 10.7759/cureus.53790

**Published:** 2024-02-07

**Authors:** Paul D Korytkowski, Oleksandr Kravtsov, Dana Hariri, Timothy A Damron

**Affiliations:** 1 Orthopedic Surgery Department, State University of New York Upstate Medical University, Syracuse, USA; 2 Pathology Department, State University of New York Upstate Medical University, Syracuse, USA

**Keywords:** musculoskeletal oncology, soft tissue tumor, ossification, calcification, ancient schwannoma

## Abstract

Benign soft tissue masses that present with atypical features on imaging may erroneously be diagnosed as malignant processes. An ancient schwannoma, a schwannoma variant with pronounced degenerative features, is one possible etiology of an incidental soft tissue tumor. This case report describes a 69-year-old male with a history of lung carcinosarcoma who presented to the orthopedic oncology office following an incidental positron emission tomography-computed tomography (PET-CT) finding of a posterior, lateral thigh mass with extensive calcifications. Subsequent excision and pathological analysis revealed an ancient schwannoma with advanced degenerative features, including metaplastic bone and cartilage formation. Various degenerative changes may typically be identified with pathological analysis. In addition to the degenerative findings on pathological analysis, our case highlights an atypical instance where extensive calcification and ossification are evident radiographically. This case emphasizes the importance of considering ancient schwannoma when a calcified soft tissue mass is encountered, in addition to the list of more common calcified soft tissue masses.

## Introduction

Schwannomas are common peripheral nerve sheath tumors. These benign entities are usually isolated findings, although they may present as multiple lesions in schwannomatosis or neurofibromatosis [1]. Schwannomas are typically asymptomatic with a low potential for malignant transformation, with malignant schwannomas comprising only approximately 6% of malignant soft tissue tumors [2]. Despite their benign nature, they have the potential to cause discomfort or neurological dysfunction. A range of histologic variants exist, including conventional, plexiform, melanotic, and cellular. The term ancient schwannoma, initially reported by Ackerman and Taylor, describes a rare schwannoma variant marked by degenerative changes. Their slow growth allows for hyalinization, hemorrhage, hemosiderin deposition, cystic degeneration, fatty degeneration, calcification, and the loss of Antoni type A cells over time [3-5]. While extensive calcification is unusual on imaging, even in ancient schwannomas, ossification is distinctly rare. Herein, we report an unusual case of ancient schwannoma of the thigh with metaplastic bone and cartilage formation resulting in extensive radiographic features. This case emphasizes that ossification, in addition to calcification, may occur in ancient schwannoma and that it must not be confused with malignant ossified soft tissue masses.

## Case presentation

**Figure 1:**
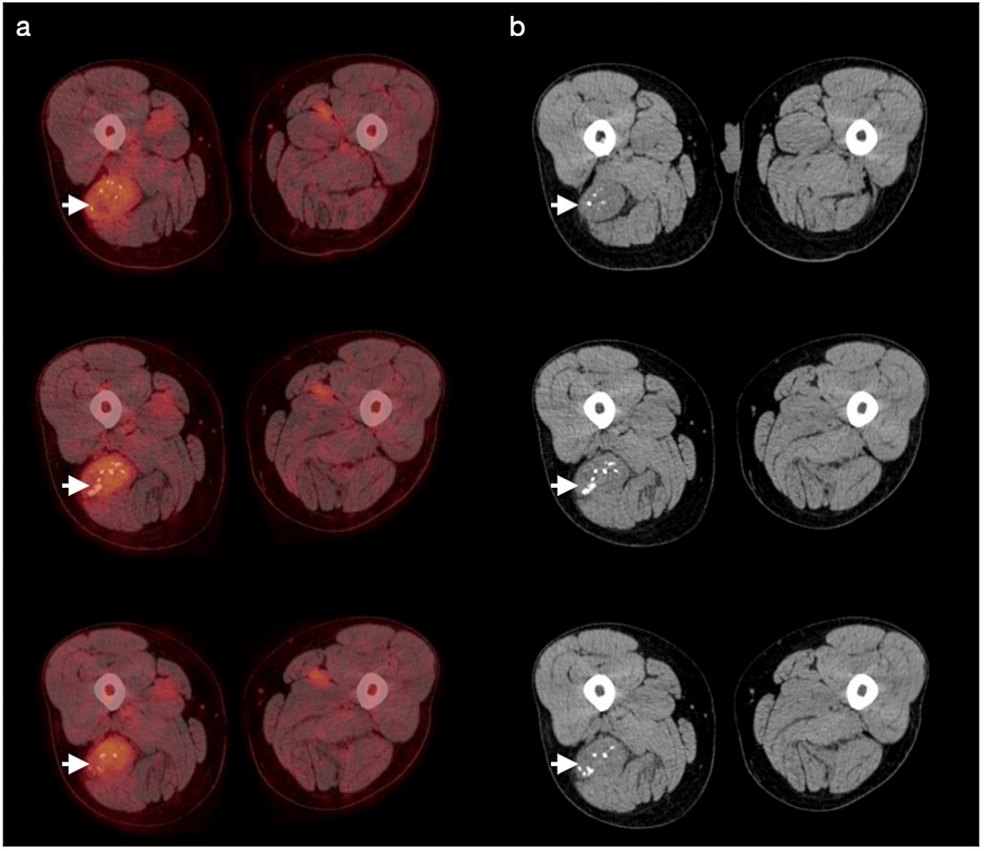
Axial images from proximal to distal (from top to bottom, respectively) showing a posterior, lateral, hypermetabolic thigh mass with extensive internal calcifications (a) Positron emission tomography. (b) Computed tomography

Informed consent was obtained from the subject according to the Institutional Review Board for the Protection of Human Subjects (IRBPHS) protocol. A 69-year-old male first presented to the orthopedic oncology office following an incidental finding of a posterior, lateral right thigh mass on imaging obtained in the emergency department at our institution following a motor vehicle collision. Computed tomography (CT) of the chest at the time revealed the incidental finding of a 2 cm spiculated right upper lobe nodule, with a subsequent biopsy showing carcinosarcoma. A staging fluorodeoxyglucose positron emission tomography (FDG-PET) scan from the skull to the mid-thigh revealed a solid 6.3 × 5.6 × 4.3 cm hypermetabolic mass in the posterior compartment of the right thigh with a maximal standard uptake value of 2.5 (Figure 1a). Extensive internal calcifications were appreciated on the corresponding computed tomography images (Figure 1b). This finding prompted concern that this carcinosarcoma was a metastatic lesion from this newly identified musculoskeletal soft tissue mass or vice versa.

Pertinent past medical history was notable for having a contralateral left axillary schwannoma excised four years prior performed by the senior author. The right thigh mass was asymptomatic and did not interfere with the range of motion or gait. Physical examination at this time showed a palpable, mildly tender, firm, deep, fixed mass in the posterior right thigh.

**Figure 2:**
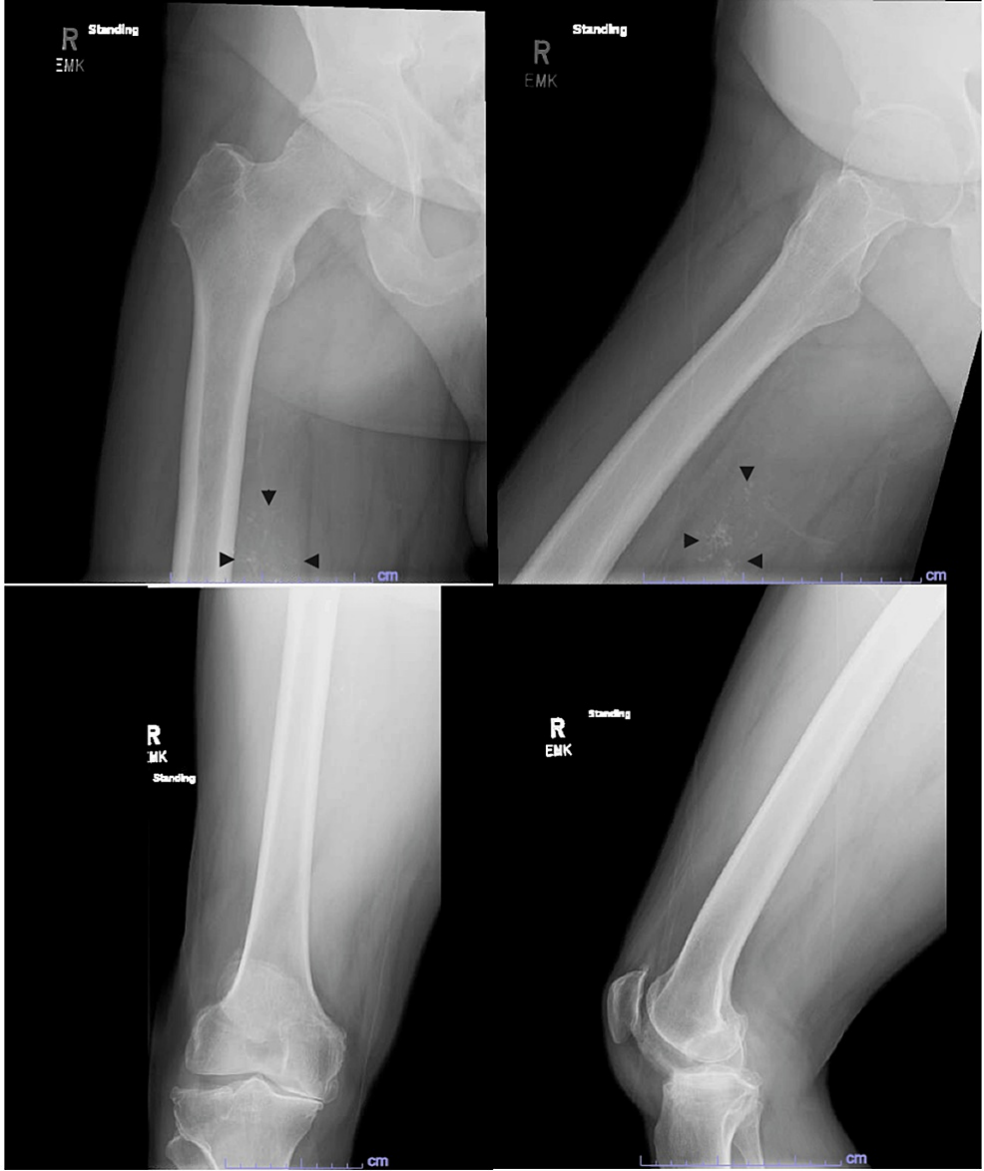
Multiple X-ray views of the femur demonstrating the calcification of a posterior soft tissue mass

**Figure 3:**
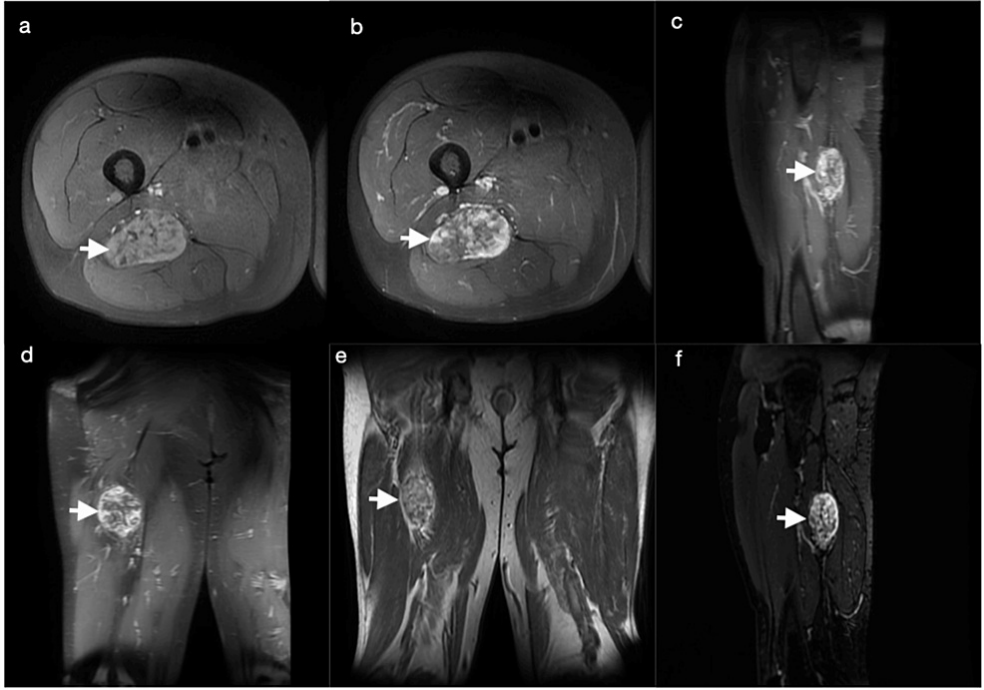
Magnetic resonance imaging of the lower extremity with and without contrast showed a well-circumscribed lesion with peripheral heterogeneous and central enhancement (a) T1; fat-suppressed; pre-contrast; TR, 793; TE, 10.36. (b) T1; axial; fat-suppressed with contrast; TR, 754; TE, 9.66. (c) T1; sagittal; fat-suppressed with contrast; TR, 810; TE, 9.40. (d) T1; coronal; fat-suppressed with contrast; TR, 697; TE, 10.09. (e) T2; bilateral coronal; TR, 4583; TE, 64.75. (f) T2; sagittal; fast spin echo; short tau inversion recovery; TR, 11532; TE, 64.51 TR, repetition time; TE, echo time

Consistent with PET-CT imaging, plain film X-rays with multiple views showed a calcified soft tissue shadow with several punctate areas (Figure 2). Magnetic resonance imaging (MRI) with and without gadobenate dimeglumine contrast showed a well-circumscribed 5.7 × 3.7 × 7.0 cm soft tissue mass with heterogeneous peripheral and central enhancement without perilesional edema (Figure 3). At this point, the differential diagnosis was broad and included all calcified soft tissue masses, including heterotopic ossification, hydroxyapatite deposition disease, hemangiomas, extraskeletal osteosarcoma, tumoral calcinosis (primary or secondary to renal insufficiency), synovial sarcoma, mesenchymal chondrosarcoma, calcinosis universalis, and connective tissue disorders such as polymyositis and dermatomyositis. Further, given the patient's history, a carcinosarcoma with calcification was considered.

Given the indeterminate findings based on imaging, the patient elected to undergo an ultrasound-guided biopsy. Initial 18-gauge core needle biopsies were nondiagnostic, demonstrating fibrin, blood, hyaline material, and focal vascular proliferation. A repeat ultrasound-guided core needle biopsy exhibited degenerated hyalinized material and focal areas of degenerative-appearing spindle cells that were positive for S100 and SOX-10 while negative for mucin-4 (MUC-4), Human Melanoma Black 45 (HMB45), cytokeratin 5/6 (CK5/6), CAM5.2, smooth muscle actin (SMA), desmin, and mouse double minute 2 (MDM2). Although the pathological results were equivocal, a benign nerve sheath tumor was suspected. Despite this, given the extent of calcifications and the ambiguity of the biopsy results, a more nefarious process, such as a malignant peripheral nerve sheath tumor, could not be excluded from the differential diagnosis. Considering the ambiguity in the diagnosis and the possibility of a malignant process, the patient opted to proceed with excision.

**Figure 4:**
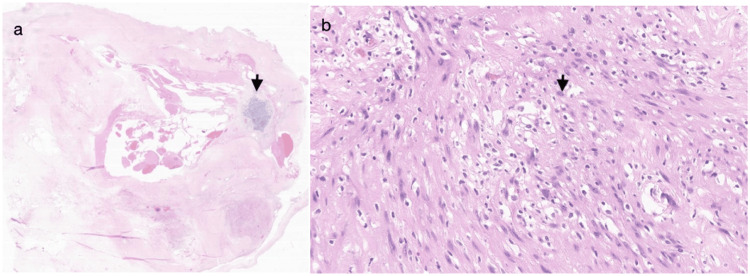
Ancient schwannoma histologic features (a) Well-circumscribed lesion consisting of predominantly hypocellular areas, focal hypercellular areas, dense hyalinization, hemorrhage, and cartilage formation (H&E, low power). (b) Focal cellular areas consisting of fascicles of bland spindle cells with clear to eosinophilic cytoplasm (H&E, high power) H&E: hematoxylin and eosin

**Figure 5:**
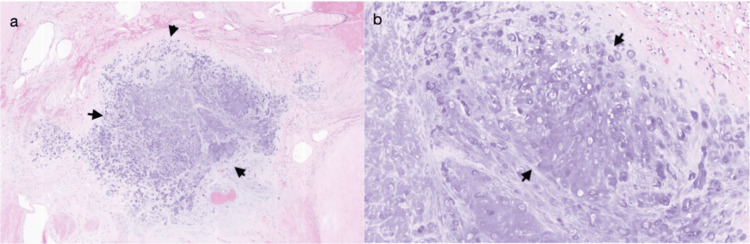
Cartilage formation H&E (a) medium power and (b) high power H&E: hematoxylin and eosin

**Figure 6:**
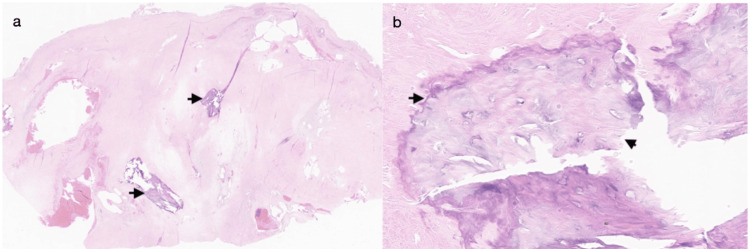
Metaplastic bone formation (a) Dense hypocellular hyalinized material with focal enlarged blood vessels and metaplastic bone formation (H&E, low power). (b) Metaplastic bone formation with lacunar spaces and osteocyte nuclei (H&E, high power). H&E: hematoxylin and eosin

A 5.5 × 5.0 × 2.5 cm, white-yellow, rubbery to partially firm and calcified, well-encapsulated, ovoid soft tissue mass with a fibrous capsule was excised. The cut surface demonstrated white-red hemorrhagic areas and was focally calcified. Histology showed an ancient schwannoma with extensive degenerative changes (Figure 4), including hyalinization, hemorrhage, cartilage (Figure 5), and metaplastic bone (Figure 6) formation. Immunohistochemical stains showed that S100 and SOX10 were positive while desmin and cytokeratin AE1/AE3 were negative, supporting the diagnosis.

## Discussion

Presented herein is the case of an ancient schwannoma with marked degenerative changes identifiable on imaging, including metaplastic bone and cartilage formation. The majority of incidental soft tissue masses identified on imaging are benign findings. As in our case, calcification and ossification identified on imaging may provide clues as to the differential diagnosis. Atypical imaging findings such as these may erroneously lead to a benign lesion being diagnosed as malignant. The mnemonic HHHOT-STCCC can be utilized to recall the differential diagnosis of calcified soft tissue masses. This includes heterotopic ossification, hydroxyapatite deposition disease, hemangiomas, extraskeletal osteosarcoma, tumoral calcinosis (primary or secondary to renal insufficiency), synovial sarcoma, mesenchymal chondrosarcoma, calcinosis universalis, and connective tissue disorders such as polymyositis and dermatomyositis. Although it is rare in many neoplasms, any soft tissue mass has the potential to calcify. Additional benign soft tissue masses that can present with calcification or ossification, potentially mimicking a malignant process, include myositis ossificans, tophaceous gout, calcific tendinopathy with osseous involvement, periosteal chondroma, primary synovial chondromatosis, Hoffa disease, lipoma with metaplasia, calcifying aponeurotic fibroma, calcific myonecrosis, ancient schwannoma, and Castleman disease [6]. The radiographic evidence of soft tissue mineralization within a schwannoma, however, is distinctly rare, particularly to the extent seen in the current case.

Although schwannomas may present anywhere, their incidence in the lower limbs is reported in only 1% of cases [7]. Radiographically, conventional schwannomas display iso- or hypo-signal intensity on T1, hyper-intensity on T2, and postcontrast enhancement. The characteristic enhancement pattern, sometimes referred to as the target sign, presents with peripheral enhancement and central hypo-intensity corresponding with the myxomatous and fibrocollagenous tissue, respectively [8,9]. Imaging findings of ancient schwannomas are uncharacteristic for schwannomas and demonstrate enhancement in the surrounding areas of degeneration and sometimes capsular enhancement. The cystic nature of the neoplasms visualized on imaging may create confusion with other diagnoses such as malignant fibrous histiocytoma, malignant peripheral nerve sheath tumor, liposarcoma, synovial sarcoma, or hemangiopericytoma [9]. However, Lee et al. described radiographic findings that may be useful in making the diagnosis, including the split fat sign, and identifying the fibrous capsule of the mass [5]. In our case, MRI revealed peripheral heterogeneous and central enhancement. The fibrous capsule was apparent and demonstrated enhancement on MRI; however, neither the target sign nor the split fat sign was present. Calcification could not be differentiated from ossification radiographically, and the pattern of mineralization was nonspecific.

Conventional schwannomas consist of hypercellular areas with fascicles or bundles of Schwann cells displaying spindle cell morphology (Antoni A pattern) or loosely textured hypocellular tissue with microcystic areas (Antoni B pattern). Parallel nuclear arrays known as Verocay bodies are common findings [1]. In a review of neurogenous tumors of the thorax, Ackerman and Taylor introduced the concept of ancient schwannomas. They identified 10 large, well-circumscribed peripheral nerve sheath tumors with features comparable to conventional schwannomas. These tumors contained fibrous nodules of diffuse overgrowth, vascularization, hyalinization, and fatty degeneration intermixed with Antoni A and B areas. The authors proposed that these degenerative changes coincided with the tumors' long duration, and the term ancient schwannoma has since been utilized to describe schwannomas with those advanced degenerative changes [4]. Calcification, an extremely rare finding in conventional schwannomas, is a well-known degenerative finding of ancient schwannomas, sometimes visible on imaging [3,10-12]. However, ossification and the extent of calcifications apparent on imaging in our case are markedly rare, which prolonged the final diagnosis even after initial biopsies.

This case focuses on very unusual radiographic findings of an ancient schwannoma that are seldom reported. The radiographic findings were due to the presence of both metaplastic bone and cartilage formation within the lesion. Ossification is a rarely reported feature of schwannomas, conventional or otherwise, with a minimal number of cases described in the literature [13-22]. These underrecognized features may be observed by pathologists with some frequency yet are sparsely reported. The radiographic manifestation of these histologic findings, however, is particularly rare, and given the nonspecific appearance, confusion in interpretation may lead to a delay in diagnosis, as it did in this case, where even the frozen-section histologic evaluation did not yield an immediately actionable diagnosis. Radiologists and clinicians from various specialties should be aware of these possible findings during radiographic evaluation and pathological analysis.

## Conclusions

In conclusion, we have presented an unusual case of ancient schwannoma with extensive degenerative findings that were identified radiographically and confirmed histologically after a review of the final formalin-fixed tissue evaluation. Our case emphasizes the importance of considering a wide array of benign and malignant soft tissue masses when atypical features, such as extensive calcification and ossification, are identified on imaging. Radiologists, orthopedic surgeons, and pathologists should be aware of these distinctive findings to inform diagnosis and clinical management.
